# Detection of *T. urartu* Introgressions in Wheat and Development of a Panel of Interspecific Introgression Lines

**DOI:** 10.3389/fpls.2018.01565

**Published:** 2018-10-29

**Authors:** Surbhi Grewal, Stella Hubbart-Edwards, Caiyun Yang, Duncan Scholefield, Stephen Ashling, Amanda Burridge, Paul Anthony Wilkinson, Ian P. King, Julie King

**Affiliations:** ^1^Nottingham BBSRC Wheat Research Centre, Plant and Crop Sciences, School of Biosciences, The University of Nottingham, Loughborough, United Kingdom; ^2^Cereal Genomics Lab, Life Sciences Building, School of Biological Sciences, University of Bristol, Bristol, United Kingdom

**Keywords:** wheat, *T. urartu*, introgression, interspecific hybrid, SNP, genetic mapping

## Abstract

*Tritcum urartu* (2*n* = 2*x* = 14, A^u^A^u^), the A genome donor of wheat, is an important source for new genetic variation for wheat improvement due to its high photosynthetic rate and disease resistance. By facilitating the generation of genome-wide introgressions leading to a variety of different wheat–*T. urartu* translocation lines, *T. urartu* can be practically utilized in wheat improvement. Previous studies that have generated such introgression lines have been unable to successfully use cytological methods to detect the presence of *T. urartu* in these lines. Many have, thus, used a variety of molecular markers with limited success due to the low-density coverage of these markers and time-consuming nature of the techniques rendering them unsuitable for large-scale breeding programs. In this study, we report the generation of a resource of single nucleotide polymorphic (SNP) markers, present on a high-throughput SNP genotyping array, that can detect the presence of *T. urartu* in a hexaploid wheat background making it a potentially valuable tool in wheat pre-breeding programs. A whole genome introgression approach has resulted in the transfer of different chromosome segments from *T. urartu* into wheat which have then been detected and characterized using these SNP markers. The molecular analysis of these wheat-*T. urartu* recombinant lines has resulted in the generation of a genetic map of *T. urartu* containing 368 SNP markers, spread across all seven chromosomes of *T. urartu*. Comparative analysis of the genetic map of *T. urartu* and the physical map of the hexaploid wheat genome showed that synteny between the two species is highly conserved at the macro-level and confirmed the presence of the 4/5 translocation in *T. urartu* also present in the A genome of wheat. A panel of 17 wheat-*T. urartu* recombinant lines, which consisted of introgressed segments that covered the whole genome of *T. urartu*, were also selected for self-fertilization to provide a germplasm resource for future trait analysis. This valuable resource of high-density molecular markers specifically designed for detecting wild relative chromosomes and a panel of stable interspecific introgression lines will greatly enhance the efficiency of wheat improvement through wild relative introgressions.

## Introduction

Common wheat has a narrow worldwide gene pool, descended from a very small number of spontaneous interspecific hybrids that originated from two natural amphiploidisation events. Domestication of wheat has further reduced its genetic variation. However, interspecific crossing with wheat’s distant wild relatives has recently been employed to overcome this genetic bottleneck (Gill et al., 2011; King et al., 2017; Zhang et al., 2017; Grewal et al., 2018). Moreover, wheat’s progenitors are being regarded as useful sources of genetic variation for many biotic and abiotic traits (Cox, 1997; Qiu et al., 2005; Börner et al., 2015; Cox et al., 2017; King et al., 2018).

*Triticum urartu* Thum. ex Gandil. (2*n* = 2*x* = 14; genome A^u^A^u^) is the A-genome donor of tetraploid wheat *T. turgidum* subsp. *durum* (2*n* = 2*x* = 42; genome AABB) and hexaploid wheat *T. aestivum* (2*n* = 2*x* = 42; genome AABBDD) (Dvorak et al., 1993) and its chromosomes are homologous to chromosomes of the A genome of bread wheat (Chapman et al., 1976). Thus, interspecific crossing between *T. urartu* and bread wheat would potentially enable transfer of desirable traits from the chromosomes of the wild diploid wheat into cultivated hexaploid wheat through direct hybridization. Previous research has shown that *T. urartu* carries many agronomically important traits, such as high net photosynthetic rate (Austin et al., 1982, 1986; Morgan and Austin, 1986) and disease resistance (Rouse and Jin, 2011; Sheedy et al., 2012), which can be exploited for improving wheat’s narrow gene pool (Qiu et al., 2005; Martín et al., 2008).

For a successful interspecific crossing program, it is vital to be able to detect the presence of and distinguish between the parental chromosomes/alleles in the hybrids. Since *T. urartu* is the donor of wheat’s A genome, traditional cytogenetic methods, such as genomic *in situ* hybridisation (GISH) and fluorescent *in situ* hybridisation (FISH), are unable to clearly distinguish between the A genome chromosomes of wheat and those of *T. urartu* in the interspecific hybrid. It also does not help that there are currently very few cytogenetic markers used for the analysis of A genome chromosomes (Adonina et al., 2015).

Previous attempts have been made at crossing *T. urartu* with other diploid wheat (Johnson and Dhaliwal, 1976; Fricano et al., 2014), tetraploid wheat (Johnson and Dhaliwal, 1976; Valkoun, 2001; Alvarez et al., 2009; Rodríguez-Suárez et al., 2011), and hexaploid wheat (Dvořák, 1976, 1978; Qiu et al., 2005). However, due to the lack of efficient cytogenetic methods for the detection of *T. urartu* chromatin in the hybrids, some studies have resorted to the use of microsatellite markers for detecting the presence of *T. urartu* alleles in wheat (Qiu et al., 2005; Rodríguez-Suárez et al., 2011). However, these markers do not provide a high-density coverage of the *T. urartu* genome. In addition, these techniques are low throughput and have limited success and are thus, not suitable for use in large-scale pre-breeding programs. Next-generation sequencing technologies and high-throughput single nucleotide polymorphism (SNP) marker development and corresponding SNP-arrays allow faster and more accurate detection of introgressions from wild relatives into wheat (Tiwari et al., 2014, 2015; King et al., 2017, 2018; Grewal et al., 2018).

In this study, we present a resource of SNP markers, spread across all seven chromosomes of *T. urartu*, which were used to identify *T. urartu* chromatin in the hexaploid wheat background. The aim of the research was to attempt to transfer chromosome segments from *T. urartu* into hexaploid wheat using a whole-genome introgression approach i.e., to exploit genetic variation from the entire genome of *T. urartu* rather than concentrate on a single introgression for a single trait, and characterize the population with a custom-designed SNP genotyping array (Winfield et al., 2016; King et al., 2017). Using these SNP markers, we were able to detect and characterize wheat-*T. urartu* recombinants which allowed us to generate a genetic map for *T. urartu* consisting of 368 SNP markers. A panel of 17 wheat-*T. urartu* recombinant lines were then selected for self-fertilization to provide a germplasm resource consisting of the whole genome of *T. urartu* introgressed into hexaploid wheat. Development of such high-density molecular markers specific for wild relative chromosomes and a panel of stable interspecific introgression lines will greatly enhance the efficiency of wheat improvement through wild relative introgressions.

## Materials and Methods

### Plant Materials

**FIGURE 1:**
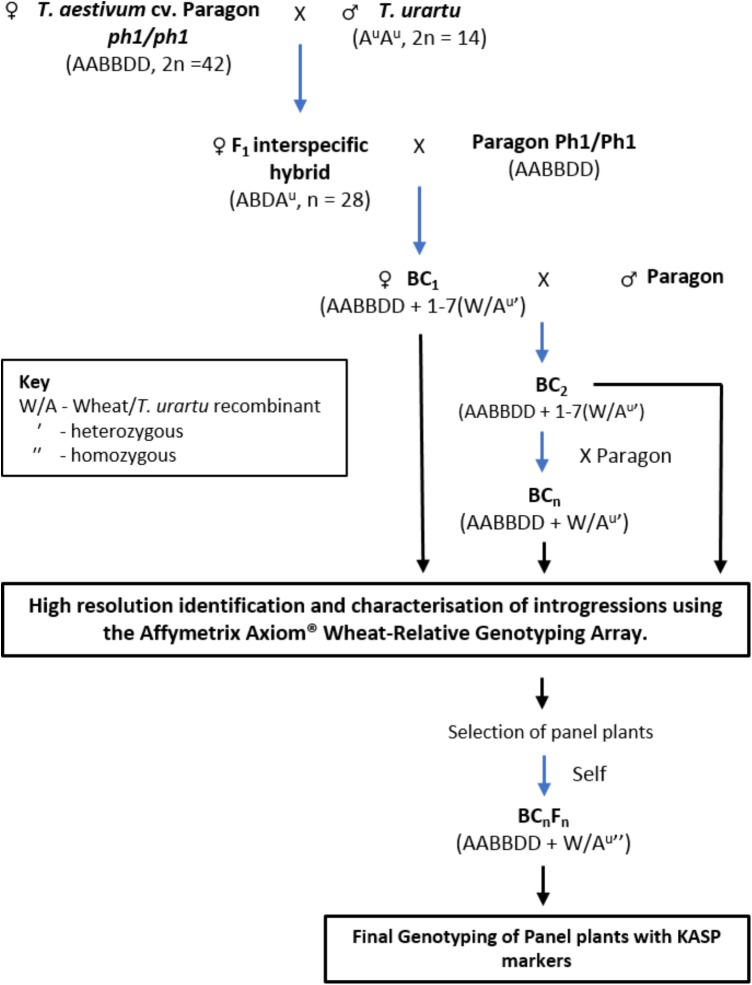
A summary of the crossing program followed to obtain interspecific wheat-*Tritcum urartu* introgression lines.

Hexaploid wheat *T. aestivum* cv. Paragon *ph1/ph1* mutant (2*n* = 6*x* = 42) was pollinated with *T. urartu* (accessions 1010001, 1010002, 1010006, and 1010020 obtained from Germplasm Resource Unit, JIC; 2*n* = 2*x* = 14) to produce F_1_ interspecific hybrids (Figure 1). The origin, according to the GRU database Seedstor, of accessions 1010001, 1010002, and 1010006 is from Armenia and that of accession 1010020 is unknown. There is no trait data available for these accessions in particular and were thus, chosen at random.

In the F_1_ hybrids, it was expected that recombination between chromosomes of *T. urartu* and wheat would occur, during gametogenesis, in absence of the *Ph1* pairing locus resulting in the production of wheat-*T. urartu* recombinants. These recombinant chromosomes would subsequently be transmitted to the progeny of these hybrid lines to generate *T. urartu* introgressions. After being grown to maturity, the F_1_ hybrids were used as the female and backcrossed with Paragon wheat, carrying the wild-type *Ph1* locus intact, to generate a BC_1_ population. The BC_1_ individuals were then recurrently pollinated with Paragon *Ph1/Ph1* to produce BC_2_, BC_3_, and BC_4_ populations (Figure 1). Three heads from each plant in each backcross population were bagged to allow self-fertilization. Cross fertility was calculated as the number of crosses setting seed.

### Genotyping via an Axiom^®^ SNP Array

To detect introgressed chromosomes and chromosome segments from *T. urartu* into wheat, an array of circa 35 K SNPs, known as the Axiom^®^ Wheat-Relative Genotyping Array (available via Thermo Fisher Scientific), was used (King et al., 2017). In summary, the array is composed of SNPs each showing polymorphisms for the ten wild relatives relative to the wheat genotypes under study. All the SNPs incorporated in this array formed part of the Axiom^®^ 820 K SNP array (Winfield et al., 2016). Detailed methods and protocols of the construction of the arrays is reported by Burridge et al. (2017). The data set for the Axiom^®^ 820 K array is available from www.cerealsdb.uk.net (Winfield et al., 2012a). This array is facilitating cost-effective, high-throughput and high resolution screening of wheat-wild relative introgressions. Table 2 shows the number of putative SNPs, for each linkage group (LG), between *T. urartu* and wheat included on the array.

The Axiom^®^ Wheat-Relative Genotyping Array was used to genotype 264 samples in total. Control samples included three replicates of each of parental lines, i.e., wheat cv. Paragon and *T. urartu* (all accessions were pooled into one sample). It should be noted that all the SNPs used on the array were also selected to be polymorphic between Paragon and all accessions of *T. urartu* used in this program. Call rate for a sample was calculated as the percentage of the number of SNP probes on the array that resulted in a definitive genotype call (AA, AB, and BB) for that sample. The equipment, software, procedures, and criteria used for this genotyping are as described by King et al. (2017).

### Genetic Mapping of *T. urartu* Chromosomes

Along with triplicates of the two parental lines, 258 lines comprising BC_1_, BC_2_, and BC_3_ populations of *T. urartu* were genotyped altogether (different generations were combined in order to have sufficient numbers of individuals) using the Axiom^®^ Wheat-Relative Genotyping Array. As described by King et al. (2017), only the Poly High Resolution (PHR) SNP markers were used for further marker analysis. PHR markers were co-dominant, polymorphic and generated minor allele calls for at least two of the three replicates of *T. urartu.* Flapjack^TM^ was used to disregard SNP markers which showed (i) heterozygous calls for either parent(s), (ii) no polymorphism between the wheat parents and *T. urartu* and/or, (iii) no calls for either parent(s) (Milne et al., 2010; v.1.14.09.24). The remaining markers were sorted into LGs in JoinMap^®^ 4.0 (Van Ooijen, 2011) with a LOD score of 30 using the genotype classification code “(a,h)”, where “a” is the genotype of the first parent and “h” is the genotype of the F_1_ hybrid. “BCpxFy” was used as the population code for each dataset which donates an advanced backcross inbred line family, where the backcross parent p had genotype “a”, x is the number of backcrosses including the one for creating the BC_1_ and y is the number of selfings, i.e., BCa1F0 is equivalent to BC_1_. The seven highest-ranking LGs were selected for downstream analysis. These were exported and assigned to chromosomes using information from the Axiom^®^ Wheat HD Genotyping Array (Winfield et al., 2012b). Erroneous markers that had more than 20% missing genotype calls were removed. LG data was used to produce a genetic map using MapChart 2.3 (Voorrips, 2002). In some cases, physical map information was employed to order loci. Graphical genotype visualization was performed using Graphical GenoTypes 2.0 (GGT; van Berloo, 2008).

### Selection of Panel Lines

After genotyping, all backcrossed lines with three or less segments introgressed from *T. urartu* were considered for construction of a panel of plants with various homozygous segments. For that purpose, a set of lines that potentially had overlapping, different sized introgressions from *T. urartu* spanning the length of each LG were selected for self-fertilization to eventually produce a panel of homozygous single segment lines that covered the entire genome of *T. urartu*.

### Comparative Analysis

Synteny analysis was carried out using sequence information of the markers located on the genetic map of *T. urartu*. The sequences of the mapped markers were used in BLAST (*e*-value cut-off of 1*e*-05) against the wheat genome IWGSC RefSeq v1.0 (Alaux et al., 2018; International Wheat Genome Sequencing Consortium [IWGSC] et al., 2018) to obtain the corresponding physical positions of the top hit in A, B, and D genomes of wheat. The sequences were also used in BLAST against the *T. urartu* reference genome sequence (Ling et al., 2018) to obtain the top hit on the Tu chromosomes. To generate the figures, map positions of the loci on the genetic map of *T. urartu* were scaled up by a factor of 100,000 to match the corresponding physical positions of the loci on the wheat A genome and the *T. urartu* (Tu) genome. Figure 5 was visualized using Circos plots (v. 0.69; Krzywinski et al., 2009) to observe (a) correlation between the markers on the genetic map of *T. urartu* and their physical positions on the Tu genome sequence and (b) synteny between the markers mapped on the A^u^ genome of *T. urartu* and the A genome of wheat. Corresponding genetic and physical positions of the markers on *T. urartu* and wheat, respectively, are shown in Supplementary Table S1.

## Results

### Generating Introgressions From *T. urartu* Into Hexaploid Wheat

A crossing program was initiated to generate gene introgressions from *T. urartu* into wheat cv. Paragon (Figure 1) using the *ph1* mutant method (Grewal et al., 2018). A total of 1902 crosses were made between wheat and *T. urartu* and their derivatives leading to the generation of 18441 crossed seed and 14193 self-fertilized seed. The number of seeds sown, germination rate, cross fertility and seed set, etc., are summarized in Table 1.

**Table 1:** Number of seeds produced and germinated in relation to the number of crosses carried out, cross fertility and the number of self-fertilized seed produced for each generation of the introgression program for *Tritcum urartu* into wheat.

	Seeds sown	Germination rate (%)	Crosses made	Cross fertility (%)	Crossed seeds produced	Seeds/Cross	Self-fertilized seeds produced
Wheat × *T. urartu*	–	–	81	40	47	0.6	–
F_1_	39	72	478	21	146	0.3	0
BC_1_	57	82	321	78	2089	6.5	10
BC_2_	204	75	754	97	11186	14.8	3163
BC_3_	301	66	243	97	3411	14	8952
BC_4_	41	91	106	100	1609	15.2	2068
Total	657	–	1902	–	18441	–	14193

Hexaploid bread wheat was used as the female parent to avoid problems with exotic cytoplasm. Sufficient viable F_1_ seeds were achieved without embryo rescue (Table 1). The F_1_ hybrids were backcrossed with Paragon wheat with the *Ph1* gene intact to generate the backcross populations. F_1_ hybrids exhibited the highest levels of infertility since they had a cross fertility of only 21% as compared to 78, 97, 97, and 100% from the crossed ears of the BC_1_, BC_2_, BC_3_, and BC_4_ generations. A further indication of the infertility of the F_1_ was shown by the fact that this generation set no self-seed in contrast to the other generations. Since the ABDA^u^ tetraploids were sterile, they were pollinated without emasculation. 478 crosses between the F_1_ hybrids and Paragon wheat resulted in 146 BC_1_ seeds. Approximately half of these BC_1_ seeds were germinated of which 43 adult plants were obtained. These BC_1_ plants also had low fertility with 24 out of 34 self-fertilized heads producing no seed. However, fertility was restored in the subsequent backcross generations.

### Molecular Marker Analysis of Wheat-*T. urartu* Introgression Lines

There are 18,287 SNPs between *T. urartu* and wheat on the Axiom^®^ Wheat-Relative Genotyping Array which were evenly spread over all seven LGs (Table 2). This array was used to screen genomic DNA prepared from 258 backcross lines between wheat and *T. urartu* along with control samples. Genotype calls were generated, and the sample call rate ranged from 83.2 to 99.9% with an average of 98.9% for the 264 samples. The lowest call rates were obtained for the three *T. urartu* samples with an average of 86.8%. Even though the Affymetrix software classified the scores for each of the probes into six cluster patterns, only those calls classified as PHR (3168) were used for genotyping as these are optimum quality.

**Table 2:** Number of SNP markers polymorphic between wheat and *T. urartu* on the Affymetrix Axiom^®^ Wheat-Relative Genotyping Array for each linkage group of the A^u^ genome and final number of SNP markers mapped onto the genetic map of the A^u^ genome of *T. urartu* obtained through Poly High Resolution (PHR) calling.

	Short Arm	Long Arm	Both Arms	% of Total SNP markers	PHR calls on genetic map	% of Total PHR calls on genetic map	cM length on genetic map
Linkage Group 1	908	1439	2347	12.8	36	9.8	92.5
Linkage Group 2	1287	1997	3284	18.0	75	20.4	147.1
Linkage Group 3	1092	1673	2765	15.1	41	11.1	108.6
Linkage Group 4	874	1294	2168	11.9	41	11.1	31.0
Linkage Group 5	686	2300	2986	16.3	81	22.0	146.0
Linkage Group 6	833	1211	2044	11.2	39	10.6	110.7
Linkage Group 7	1320	1373	2693	14.7	55	14.9	136.1
Total	7000	11287	18287	100.00	368	100.0	772.1

**FIGURE 2:**
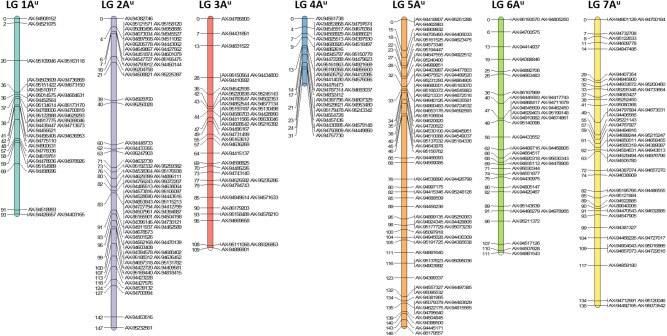
Genetic linkage map of *T. urartu* showing 368 SNP markers spread across seven linkage groups (LGs).

After filtering out 2509 good quality PHR SNPs using Flapjack^TM^, JoinMap^®^ was used to genetically map the markers by analyzing the corresponding genotypes of all lines. In order to get strongly linked loci a high LOD score was used which led to the establishment of seven LGs that were composed of 368 SNPs and represented the seven chromosomes of *T. urartu* (Figure 2). Within the mapped PHR SNPS, LG 5 had the highest number of SNPs (22%) while LG 1 had the lowest (9.8%). A genetic map was constructed (Figure 2) with a total map length of 772.1 cM (Table 2) and an average chromosome length of 110.3 cM. It should be noted that the germplasm used to generate these linkage maps did not constitute proper mapping populations and in fact we combined different generations in order to have sufficient numbers. Therefore, the cM distances in the map generated should be treated with considerable caution. However, the map did allow the ordering of the markers and hence, the identification and tracking of segments through backcross generations.

### Detection of Introgressions and Panel Selection

**FIGURE 3:**
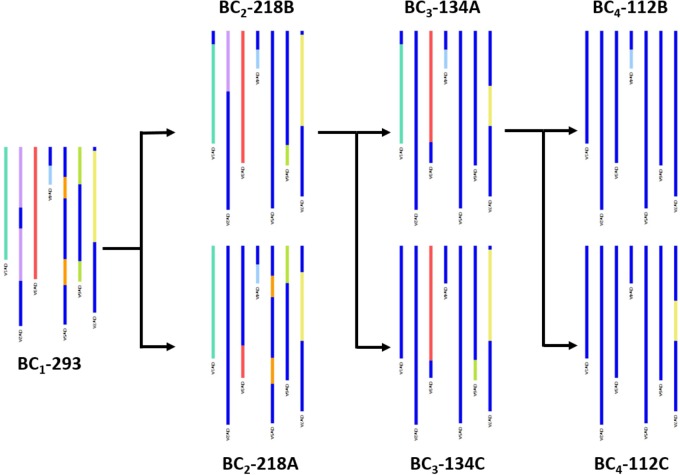
A graphical representation of SNP characterisation of *T. urartu* introgressions into a hexaploid wheat background in four consecutive generations, i.e., BC_1_, BC_2_, BC_3_, and BC_4_, through GGT bar diagrams. Wheat genotype is represented in dark blue and *T. urartu* genotype is represented in a different color for each LG.

In Figure 3, an example of how the genetic map allowed the tracking of *T. urartu* introgressions, through the backcrossed populations, is shown. Presence of *T. urartu*, shown in colored segments, could be visualized through GGT bar diagrams which allowed the graphical representation of the genotyping data for each line, i.e., the markers on the genetic map. The dark blue region of the GGT bars represent the wheat allele for a marker. Introgressions could be tracked from the BC_1_ plant (BC_1_-293), which carried *T. urartu* segments from each of the seven LGs, through to the single segment BC_4_ lines (BC_4_-112B and BC_4_-112C). Of the two BC_2_ plants (BC_2_-218A and BC_2_-218B) originating from the BC_1_ plant, both carrying segments from six *T. urartu* LGs, BC_2_-218B was propagated further to produce two BC_3_ plants, BC_3_-134A and BC_3_-134C. The former was further backcrossed to produce two BC_4_ plants, each with a different *T. urartu* segment.

**FIGURE 4:**
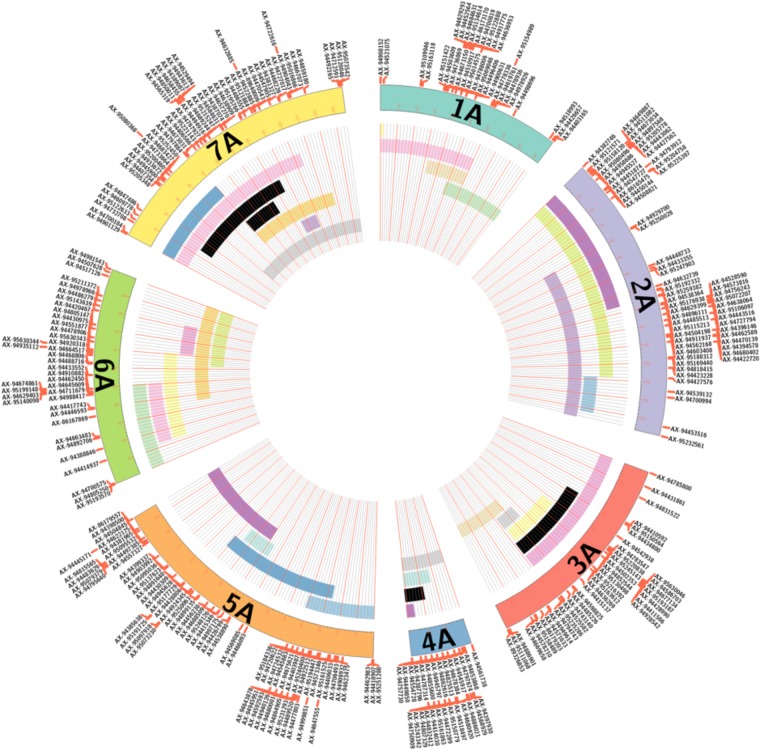
A graphical representation of various sized segments of *T. urartu*, along its linkage map, in introgression lines involved in panel selection. For each of the seven LGs, the map positions of the corresponding molecular markers are reported. Black bars represent single chromosome segments of *T. urartu* detected in the introgression lines, while bars with the same color indicate multiple chromosome segments of *T. urartu* detected in a single introgression line. In total, 17 lines with overlapping segments that covered the entire genome of *T. urartu* were selected for a panel of lines that would undergo self-fertilization for downstream trait analysis.

Furthermore, all lines with 3 or less segments from *T. urartu* were considered for self-fertilization. From these, 17 lines were selected which had a combination of introgressed segments that would overlap to cover the entire genome of *T. urartu* as shown in Figure 4 and Supplementary Table S2. In lines with multiple segments, each segment is color-coded with the same color, i.e., *T. urartu* segments in different LGs of the same color in Figure 4 belong to one introgression line. This panel of *T. urartu* introgression lines, where each line contained between 1 and 3 segments, are currently being self-fertilized for downstream trait analysis.

### Comparative Analysis of Wheat and *T. urartu* Genomes

**FIGURE 5:**
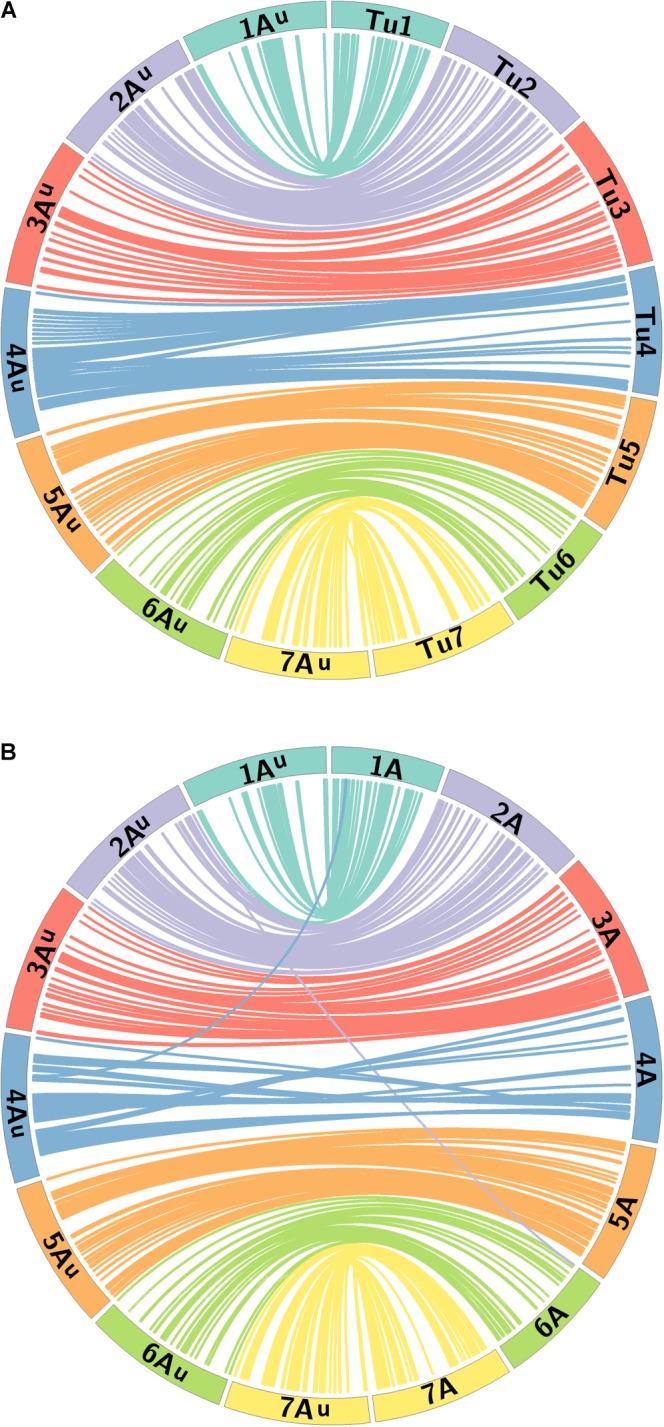
Comparative analysis between the A^u^ genome loci on the genetic map of *T. urartu* and their physical positions on **(A)** the Tu chromosomes and **(B)** the A genome of wheat. Homeologous chromosomes from 7 LGs between *T. urartu* and wheat are displayed as ideograms of the same color. Lines connect genetic map positions of markers in the A^u^ genome (left of plot) to their physical map positions (right of plot) on the **(A)** Tu chromosomes and **(B)** the A genome of wheat. Lines joining A^u^ and wheat A chromosomes with the same color as the ideograms indicate synteny through mapping in homeologous LGs. Lines between the A^u^ and the wheat A genome that end in a different colored ideogram in the wheat genome point to mapping in non-homoeologous LGs.

A BLAST analysis of the 368 marker sequences on the A^u^ genome map against their physical positions on the *T. urartu* genome (Tu chromosomes) indicated that the order of markers on the genetic map correlates well with their physical order on the Tu chromosomes. 341 markers resulted in a BLAST hit against the Tu genome sequence. Figure 5A shows that the seven LGs of mapped markers on the A^u^ genome also map back to their corresponding Tu chromosome group, i.e., markers in LG 1 had a BLAST top hit on chromosome Tu1, and the markers are well distributed on each of the seven Tu chromosomes.

A macro-colinearity analysis was carried out to determine any occurrences of major chromosome rearrangements in the A genome during or after the formation of hexaploid wheat. Marker sequences on the genetic map of *T. urartu* were also used in BLAST analysis against the wheat Chinese Spring genome assembly. Physical position for the top hit from the A genome of wheat, where available, and for the overall top hit (maximum sequence identity match) for either of the 3 wheat genomes was obtained (Supplementary Table S1). The BLAST results showed that 92.4, 74.7, and 76.4% of the markers had a significant BLAST hit on the A, B, and D genomes of wheat, respectively. Of these BLAST hits, 73.9, 13.6, and 19.8% of the markers had an overall top hit on the A, B, and D genomes of wheat, respectively, with some showing the same score for the top hit for more than one genome.

Figure 5B shows the syntenic relationship between the seven LGs of the A^u^ genome of *T. urartu* and the A genome of wheat with colored lines showing significant synteny and collinearity. Some gene rearrangements are indicated where single markers cross map to positions on non-homeologous wheat chromosomes. The only major disruption in collinearity between the two species is that the wheat chromosome 4A has an inversion compared to *T. urartu* chromosome 4A^u^. The latter has the 4/5 translocation like wheat but does not carry the 4/7 translocation observed for chromosomes 4A and 7B of wheat (Liu et al., 1992; Devos et al., 1995). This data demonstrates the close syntenic relationship between the A genome of wheat and *T. urartu*.

## Discussion

*Tritcum urartu* is a potentially important source of genetic variation for a wide variety of agronomically important traits (Austin et al., 1982; Qiu et al., 2005; Martín et al., 2008; Rouse and Jin, 2011; Sheedy et al., 2012). Using the *ph1* mutant approach, wheat-*T. urartu* recombinant lines have been generated in this study (Figure 1) suggesting that recombination can occur between hexaploid wheat and *T. urartu* chromosomes. Similar crossing strategies have been used previously to generate wheat-wild relative recombination (Grewal et al., 2018). Moreover, a high rate of recombination allowed generation of a genetic map for *T. urartu* indicating that the chromosomes of *T. urartu* and the A genome of bread wheat have high homology.

Cross fertility is lowest in the F_1_ hybrids at 21% but increases substantially in the back-cross generations reaching 100% in the BC_4_ population (Table 1). This was expected since the inter-specific F_1_ hybrids were haploid for the A, B, D, and A^u^ genomes and the frequency of recombination between chromosomes from different genomes is likely to be very low leading to unviable gametes. However, cross fertility and seeds set per cross increase remarkably in the backcross generations and self-fertility is restored after only two backcrosses.

Traditional cytogenetic methods such as GISH are not helpful in detecting the presence of *T. urartu* chromatin in the wheat background in an interspecific hybrid since *T. urartu* is the A genome donor of common wheat (Dvorak et al., 1993). In addition to traditional FISH probes such as pSc119.2 and pAs1, probe pTm30, essentially a (GAA)n microsatellite marker, has been shown to produce major hybridization sites on the A genome chromosomes of diploid wheats including *T. urartu* (Adonina et al., 2015). However, these FISH probes are still not able to distinguish between all A genome chromosomes and they demonstrate polymorphisms between accessions of different diploid and hexaploid wheats (Adonina et al., 2015). Moreover the (GAA)n microsatellite marker has been shown to distinguish between A genome chromosomes of diploid wheats such as *T. urartu*, *T. boeticum*, and *T. monococcum* but not between the A genome chromosomes of *T. urartu* and hexaploid wheat (Megyeri et al., 2012; Adonina et al., 2015). This makes detection of chromosomes originating from wild diploid A genome species, such as *T. urartu*, difficult in the presence of the A genome chromosomes of hexaploid wheat.

In the absence of clear cytogenetic characterisation of wheat-*T. urartu* introgression lines, SNP markers prove vital in enabling the detection of *T. urartu* chromosomes in a wheat background. The Axiom^®^ Wheat-Relative Genotyping Array has been successfully validated as a high throughput genotyping platform consisting of SNP markers that are able to detect the presence of various wheat wild relatives in a hybrid line (King et al., 2017, 2018; Grewal et al., 2018). In previous studies that have used this array, the introgressions detected by the SNP markers were also validated by GISH studies thereby indicating that the array was successful at detecting the presence of various sized segments of wild relatives in a wheat background. In this study using the same array, 368 SNPs were mapped into seven LGs that represented the genetic map of *T. urartu* with a total map length of 772.1 cM (Figure 2). The average chromosome length was found to be 110.3 cM, however, for chromosome 4A^u^ it was calculated to be 31 cM (Table 2) due to the least number of recombination events in this LG as compared to the others. This was possibly due to the rearrangement of wheat chromosome 4A (Devos et al., 1995) which impacted the recombination between chromosomes 4A and 4A^u^. This result is supported by the comparative analysis of the markers on the genetic map of textitT. urartu and their orthologous sequences on the Tu chromosomes and the wheat A genome (Figure 5). The comparison of the chromosomes showed high levels of collinearity and synteny between the two species, including the presence of the 4A/5A translocation in *T. urartu* which has been previously reported (King et al., 1994), except in LG 4 where the wheat chromosome 4A showed an inversion as compared to chromosome 4A^u^. However, it should be noted that the SNP markers described in this paper are not able to distinguish which of the genomes of wheat the *T. urartu* introgressions have recombined with. The introgressions were produced using the *ph1* system and therefore it is possible that recombination has taken place between the *T. urartu* and the B or D genomes of wheat as well as the A genome. It is possible to use multi-color GISH to distinguish the A, B and D genomes of wheat (King et al., 2017; Grewal et al., 2018) and thus, visualize an A-B or A-D recombinant. However, because of the *ph1* system used in this work, it is possible that recombination could have also occurred between the three genomes of wheat. It would therefore be impossible to determine which A genome (A or A^u^) was involved in any recombination event with the B or D genomes of wheat. We are currently developing a set of wheat genome specific markers which will enable the identification of the wheat genome involved in the recombination once the introgression lines are homozygous and stable, i.e., these markers would be able to detect which of the wheat genome regions had been replaced by the *T. urartu* introgressions.

Through marker assisted selection, the *T. urartu* segments were tracked in the backcross populations (Figure 3) leading to identification of lines with three or fewer segments that were eventually self-fertilized. From these lines, a panel of 17 interspecific lines, having various sized introgressions that potentially span the entire genome of *T. urartu*, is also described in this study (Figure 4). These lines aim to provide a valuable germplasm resource for phenotyping program, with the aim of transferring a wide variety of traits from *T. urartu* into all regions of the wheat genome for the introduction of genetic variation.

## Data Availability

The raw genotyping data supporting the conclusions of this manuscript will be made available by the authors, without undue reservation, to any qualified researcher.

## Author Contributions

JK, SG, CY, SH-E, DS, SA, and IK carried out the crossing program. SH-E, DS, SA, and CY prepared the samples for genotyping. AB ran the samples on the array. SG analyzed the genotyping data and constructed the genetic map. SG and PW worked on the comparative studies. IK and JK conceived and designed the experiments. SG wrote the manuscript with assistance from JK. All authors have read and approved the final manuscript.

## Conflict of Interest Statement

The authors declare that the research was conducted in the absence of any commercial or financial relationships that could be construed as a potential conflict of interest.
